# The Book World of Medicine and Science

**Published:** 1895-12-21

**Authors:** 


### The Book World of Medicine and Science.

Diet in Sickness and in Health. By Mrs. Ernest Hart, with an introduction by Sir Henry Thompson, F.R.C.S. (London: The Scientific Press. 1895. Pp. 219.)

It is certainly an interesting book, and in its way a clever one. As Sir Henry Thompson says in his introduction, the first thing necessary for "food and feeding in health and disease" is to be well instructed in the elements of physiology; the second, "some practical acquaintance with kitchen usage and processes." Herein lies Mrs. Hart's great advantage as a teacher on this subject; she is not only a physiologist, but has gained a "long culinary and housewifery experience." A fair general sketch is given of the general principles of the physiology of digestion, which will be found very useful by many, although we may doubt as to the great advantage which is likely to accrue to the various classes of readers to whom the book is addressed from the anatomical and pathological illustrations which are interspersed in the text. There can be no doubt that the practical portions of the book are the best; and where Mrs. Hart speaks out of her own knowledge, she not only speaks with the authority derived from experience, but with the ease and freedom of an expert. We need not be surprised to find the virtues of the non-alcoholic stimulants or restoratives—we like the word restoratives—sung with much force; and, indeed, much evidence is given of the power of coca, tea, coffee, &c., to enable people to get through great bodily or mental work when it is necessary to make a spurt. No small art is displayed in the "setting out" of the contents of the book, and if it had been published by a struggling physician one might fairly prophesy a large clientèle from the heading of two chapters alone. "Thinning the Fat" and "Fattening the Thin" should afford work for a lifetime to any man, together with no mean profit. It is interesting, and shows well the practical nature of Mrs. Hart's observations, to find her stating that the gouty person should not be a vegetarian, and that on the contrary his diet "should be limited to beef, mutton, chicken, game, fish, eggs, green vegetables, a few ounces of stale bread, and a small quantity of butter." This is not bad, and on it one feels content to face even a gouty old age. Truly Mrs. Hart says that the gouty person should never eat to satiety, but as the dictionary defines satiety as "fulness of gratification beyond desire or pleasure," one does not greatly mind. Probably the truth lies in the injunction to take "active exercise." Nevertheless, it is not without interest to observe, even in so outspoken a teacher as Mrs. Hart, a tendency to "hedge," for the next chapter gives due prominence to the views of Dr. Haig, showing the many ills which are due to the accumulation of uric acid, and how they, including gout, are to be treated by a vegetable diet. The true mean, however, is properly struck by Mrs. Hart when she says that the gouty person must be an ascetic in the matter of eating and drinking, and that water is the only article of diet the gouty may take in excess. In addition to the advantages of fresh air and exercise, Mrs. Hart very rightly points out the benefit derived from residence in a warm, dry, equable climate, and, speaking of Grand Canary and Teneriffe, she hazards the conjecture that it will not be long before the martyrs to arthritic gout will learn that the "Fortunate Islands" may be to them a discovery worth making. Certainly the most practically important part of the book is that devoted to diabetes, and we expect that these chapters will be read with interest not only by medical men and by those suffering from diabetes proper, but by the very large class of people who are afflicted with that false and much more amenable form of sugar production which is associated with dyspepsia. We have perused this book with great pleasure, and feel sure that many will find in it much to lighten the burden of illness and to lengthen the days of those whose digestion is enfeebled.

**Elementary Physiology. Blackie's Science Text-Books.** By J. R. Ainsworth Davis, B.A. (London: Blackie and Son. 1895. 2s.)

This little primer will be found very easy reading by the merest tyro; it appears to us to be especially adapted for the physiology classes of young ladies' academies or boys' schools, since it contains no rude truths that would shock the most susceptible of parents or satisfy the prurient curiosity which often leads the younger sort to take an interest in the study of life. The book is well illustrated with a number of diagrams, which, if not original, are at least new to works on physiology. A section on the secretion of milk and the digestion in the cow may be of use to agricultural students or to those interested in dairy work. The author has, we must admit, condensed into 200 small pages of large type the main features of general physiology, a feat for which we give him our best congratulation.

